# Polymorphous low-grade neuroepithelial tumor of the young (PLNTY): an epileptogenic neoplasm with oligodendroglioma-like components, aberrant CD34 expression, and genetic alterations involving the MAP kinase pathway

**DOI:** 10.1007/s00401-016-1639-9

**Published:** 2016-11-03

**Authors:** Jason T. Huse, Matija Snuderl, David T. W. Jones, Carole D. Brathwaite, Nolan Altman, Ehud Lavi, Richard Saffery, Alexandra Sexton-Oates, Ingmar Blumcke, David Capper, Matthias A. Karajannis, Ryma Benayed, Lukas Chavez, Cheddhi Thomas, Jonathan Serrano, Laetitia Borsu, Marc Ladanyi, Marc K. Rosenblum

**Affiliations:** 1 Departments of Pathology and Translational Molecular Pathology, University of Texas MD Anderson Cancer Center, 2130 W Holcombe Blvd, LSP9.4009, Houston, TX 77030 USA; 2 Department of Pathology, New York University Langone Medical Center, New York, NY 10016 USA; 3 Division of Pediatric Neuro-oncology, German Cancer Research Center (DFKZ), 69120 Heidelberg, Germany; 4 Department of Pathology, Nicklaus Children’s Hospital, Miami, FL 33155 USA; 5 Department of Radiology, Nicklaus Children’s Hospital, Miami, FL 33155 USA; 6 Department of Pathology and Laboratory Medicine, Weill Cornell Medical College, New York, NY 10065 USA; 7 Murdoch Children’s Research Institute, Royal Children’s Hospital, Parkville, VIC 3052 Australia; 8 Institute of Neuropathology, University of Erlangen, 91054 Erlangen, Germany; 9 Department of Pathology, University of Heidelberg, 69120 Heidelberg, Germany; 10 Department of Pediatrics, New York University Langone Medical Center, New York, NY 10016 USA; 11 Department of Ototlaryngology, New York University Langone Medical Center, New York, NY 10016 USA; 12 Department of Pathology, Memorial Sloan-Kettering Cancer Center, 408 E 69th St. (Z564), New York, NY 10065 USA

**Keywords:** Low-grade neuroepithelial tumor, Epilepsy, Oligodendroglioma, BRAF, FGFR2, FGFR3

## Abstract

Epileptogenic tumors affecting children and young adults are a morphologically diverse collection of neuroepithelial neoplasms that, as a group, exhibit varying levels of glial and/or neuronal differentiation. Recent advances in molecular profiling technology, including comprehensive DNA sequencing and methylation analysis, have enabled the application of more precise and biologically relevant classification schemes to these tumors. In this report, we describe a morphologically and molecularly distinct epileptogenic neoplasm, the polymorphous low-grade neuroepithelial tumor of the young (PLNTY), which likely accounts for a sizable portion of oligodendroglioma-like tumors affecting the pediatric population. Characteristic microscopic findings most notably include infiltrative growth, the invariable presence of oligodendroglioma-like cellular components, and intense immunolabeling for cluster of differentiation 34 (CD34). Moreover, integrative molecular profiling reveals a distinct DNA methylation signature for PLNTYs, along with frequent genetic abnormalities involving either B-Raf proto-oncogene (*BRAF*) or fibroblast growth factor receptors 2 and 3 (*FGFR2*, *FGFR3*). These findings suggest that PLNTY represents a distinct biological entity within the larger spectrum of pediatric, low-grade neuroepithelial tumors.

**Electronic supplementary material:**

The online version of this article (doi:10.1007/s00401-016-1639-9) contains supplementary material, which is available to authorized users.

## Introduction

A diverse array of low-grade neuroepithelial tumors (LGNTs) has been associated with epilepsy in children and young adults. These neoplasms exhibit varied histopathologic features and glial or glioneuronal differentiation, recognized types including pilocytic astrocytoma, diffuse astrocytoma (DA), ganglioglioma, pleomorphic xanthoastrocytoma (PXA), dysembryoplastic neuroepithelial tumor (DNET), angiocentric glioma, and oligodendroglioma [26]. Recent large-scale molecular profiling studies have established the genomic landscape characterizing the broad spectrum of LGNTs, while also pointing toward more molecularly uniform disease subgroupings [31, 48]. In particular, genome-wide DNA methylation profiling and the identification of recurrent genomic aberrations (e.g., mutations, rearrangements, and copy number abnormalities) have been crucial in the delineation of biologically distinct disease entities and in refining tumor classification.

Here, we describe a unique epileptogenic LGNT variant affecting children and young adults, which we call polymorphous low-grade neuroepithelial tumor of the young (PLNTY). We show that PLNTYs, while morphologically variable, are uniformly characterized by the presence of oligodendroglioma-like cellular components, infiltrative growth patterns, and intense CD34 immunopositivity. Moreover, we demonstrate that PLNTYs exhibit a distinct DNA methylation signature, most closely related to that of ganglioglioma, and harbor molecular abnormalities involving mitogen-activated protein kinase (MAPK) pathway constituents. Our findings suggest that PLNTYs may represent a distinct biological entity within LGNTs of children and young adults.

## Materials and methods

### Histopathology

Case materials were collected by the authors in the course of their hospital or consultative (MKR) practices. Formalin-fixed and paraffin-embedded (FFPE) tissues were employed for all immunohistochemical and molecular diagnostic assays. The streptavidin–biotin peroxidase complex method was utilized for immunohistochemical studies with antibodies to: glial fibrillary acidic protein (GFAP; Biogenex, polyclonal), synaptophysin (SYN; Biogenex, monoclonal), neuronal nuclear protein (NeuN; Chemicon, monoclonal), neuronal protein HuC/HuD (HuC/HuD; Molecular Probes, monoclonal), chromogranin (CHR; Ventana, monoclonal), epithelial membrane antigen (EMA; Sigma/Aldrich, monoclonal), CD34 (Ventana, monoclonal), Ki-67 (MIB-1; Ventana, monoclonal), isocitrate dehydrogenase 1 (IDH1) R132H (Dianova, H09 clone, monoclonal), BRAF V600E (Spring Bioscience, monoclonal), α-thalassemia mental retardation X-linked (ATRX; Sigma, polyclonal), and oligodendrocyte lineage transcription factor 2 (OLIG2; Millipore, polyclonal).

### Mutational genotyping

BRAF V600E mutations were detected using the Sequenom Mass ARRAY system as previously described [1].

### Fusion transcript detection

Total RNA extracted from FFPE material was analyzed for fusion transcripts using anchored multiplex PCR (AMP)-based methodology (ArcherDx) [49]. Briefly, unidirectional gene-specific primers (GSPs) were used to target specific exons in 35 genes frequently involved in chromosomal rearrangements. GSPs, in combination with adapter-specific primers, enriched for known and novel fusion transcripts. 220 GSPs ranging from 18 to 39 base pairs in length were provided by Archer and are designed either in the 5′ or 3′ direction of the corresponding gene.

### Genome-wide DNA methylation profiling

DNA methylation was performed at the NYU molecular laboratory and the Genomics and Proteomics Core Facility of the German Cancer Research Center (DKFZ) using the Illumina Infinium HumanMethylation450 (450 k) array assessing 482,421 CpG sites (Illumina, San Diego, USA), according to the manufacturer’s instructions. DNA methylation data were normalized by performing background correction and dye bias correction (shifting of negative control probe mean intensity to zero and scaling of normalization control probe mean intensity to 10,000, respectively). Filtering of probes included removal of probes targeting the X and Y chromosomes (*n* = 11,551), removal of probes containing a single-nucleotide polymorphism (dbSNP132 Common) within five base pairs of and including the targeted CpG-site (*n* = 24,536), and probes not mapping uniquely to the human reference genome (hg19) allowing for one mismatch (*n* = 9993). To enable future comparability, we also removed probes not represented on the updated Illumina Infinium HumanMethylationEPIC array. In total, 428,799 probes were kept for analysis. For unsupervised hierarchical clustering, we selected the 2500 probes that showed the highest standard deviation across the beta values. Samples were hierarchically clustered using 1—Pearson correlation as a distance measure and average linkage as agglomeration method, with unscaled methylation levels shown in a heat map from unmethylated state (blue color) to methylated state (red color). Copy number profiles were generated using the ‘conumee’ R package in Bioconductor (http://www.bioconductor.org/packages/release/bioc/html/conumee.html) and assessed manually for regions of interest.

## Results

### PLNTYs occur in younger patients in association with epileptogenic activity, and tend to exhibit a benign (WHO grade I) clinical course

We assembled demographic and clinical data for ten patients with PLNTYs whose material was examined in the Department of Pathology at MSKCC (Table 1). Six patients were females and four were males, aged 4-32 (mean = 17.6, median = 17.5) years at tissue diagnosis. Only four patients were older than 18 years of age and one of these, a 32 year-old female representing the oldest patient in the cohort at diagnosis, had tumor-related symptoms (temporal lobe seizures) since 6 years of age. Save for one patient (case 8) who reportedly had prior resection of a meningioma 18 years previously, no patient had a history or evidence of another neoplastic or syndromic disorder. Excepting two patients with occipital lobe lesions, including one patient (case 7) who had headaches and dizziness of six days duration and one patient (case 10) who had a two-year history of visual disturbances, all patients suffered refractory epilepsy of 1-20 years duration prior to diagnosis.

**Table 1:** Demographic, clinical, and molecular features of PLNTYs

Case #	Age at Dx (years)	Gender	Clinical presentation	Duration	Location	Treatment	Follow-up (months)	Outcome	BRAF mutation	Fusion transcripts
1	16	M	Seizures	>2 years	R med temporal	GTR	53	NED, no seizures	BRAF V600E	
2	18	F	Seizures	1 year	R med temporal	STR	18	Post-op psychosis, now SD	BRAF V600E	
3	23	F	Seizures	Unknown	R temporal	N/A	N/A	N/A	BRAF V600E	
4	17	F	Seizures	Unknown	R temporal	GTR	89	NED, no seizures	Neg	FGFR3-TACC3
5	4	M	Seizures	Unknown	L temporal	GTR	86	NED, no seizures	Neg	FGFR2-CTNNA3
6	9	M	Seizures	6 years	R frontal	GTR	36	New seizures and MRI abnormality after 36 months	Neg	FGFR2-KIAA1598^a^
7	10	M	HA/dizziness	6 days	R occipital	GTR	62	NED	Neg	FGFR2-KIAA1598
8	23	F	Seizures	N/A	R medial temporal	GTR	12	NED	Neg	
9	32	F	Seizures	26 years	R temporal	GTR	55	NED, no seizures	N/A	
10	24	F	Visual disturbances	2 years	R occipital	GTR	12	NED	N/A	

Cases 1–8 received BRAF genotyping and methylation profiling. Cases 1–5, and 7 received ArcherDx profiling

^a^Presence of fusion inferred by deletion event chromosome 10

**Fig. 1:**
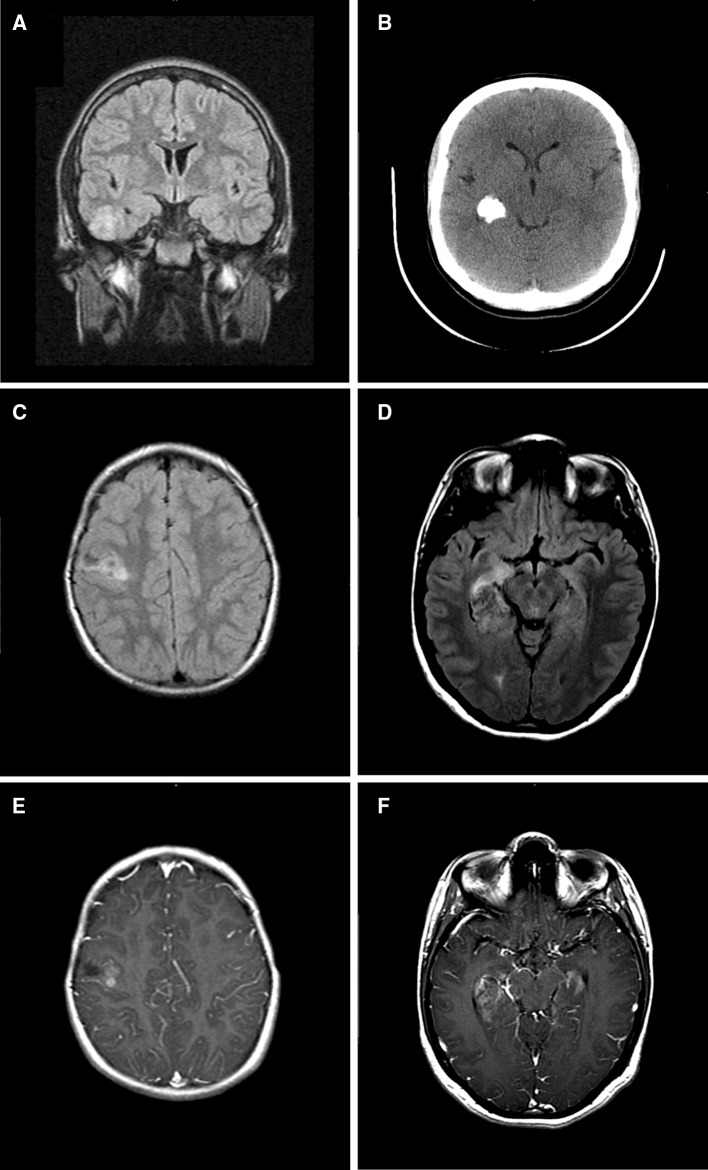
Radiographic features of PLNTY. Typical appearance of a unifocal abnormality best seen on FLAIR sequence (**a**; case 1). Frequent calcifications appreciated on CT scan (**b**; case 2). Focal contrast enhancement was seen in two cases (**c**–**f**) as demonstrated by FLAIR (**c**, **d**) and T1 post-contrast (**e**, **f**) images for cases 6 (**c**, **e**) and 2 (**d**, **f**). Cystic change was also seen in case 6 (**c**, **e**)

Seven tumors were situated in the temporal lobes (six right and one left), four of which were medially positioned. One of the latter (case 2) extended into the posterolateral thalamus and posterior limb of the internal capsule (PLIC). Two of three extratemporal tumors were in the right occipital lobe and one was in the right frontal lobe. In total, nine of ten lesions were right-sided. Neuroradiologic studies were available for review in seven cases. Features that would distinguish the lesions under consideration from other primary brain tumors or space-occupying processes characteristic of this age group were not identified. All presented as unifocal regions of FLAIR hyperintensity with increased or mixed signal characteristics on T_2_—weighted imaging. Maximal recorded dimensions ranged from 0.7 to 3 cm. Four exhibited cystic components. Only two cases (2 and 6) manifested areas of irregular or nodular enhancement in post-gadolinium T_1_—weighted studies (Fig. 1a–d) and none were associated with significant mass effect or edema. Save for case 2, a tumor centered in the right medial temporal lobe with extension into the posterolateral thalamus and PLIC, all lesions were superficially situated. Three patients had CT studies, all demonstrating lesion-associated calcifications. In two cases (1 and 2), tumors remained stable in size over surveillance intervals of 12 and 20 months. A third lesion (case 10) demonstrated enlargement from 0.7 to 0.9 cm in greatest dimension over 24 months of observation while a fourth (case 6) remained stable over 48 months of surveillance and enlarged slightly over an additional 24 months. Neuroradiologic diagnoses included glioma, oligodendroglioma, DNET, and focal cortical dysplasia.

Details of treatment and outcome were available for nine patients (Table 1). Follow-up intervals ranged from 12 to 89 (mean = 47) months, with five patients monitored longer than 50 months from diagnosis. Of eight patients who had gross total tumor resections, all were free of disease at last follow-up save for patient six who suffered breakthrough seizures 36 months after surgery and was found to have a possibly new area of FLAIR signal abnormality at the base of the resection cavity. Of the five other patients with refractory epilepsy who had gross total excisions, four were rendered seizure-free and one had a marked reduction in seizure frequency. The single patient (case 2) who underwent subtotal resection for a tumor extending from the temporal lobe to the thalamus and PLIC had stable radiographic disease at 18 months but had developed psychotic manifestations (hearing voices, exhibiting hyper-religiosity, and aggressive behavior) and attempted suicide.

### Despite histopathologic variability, PLNTYs are invariably characterized by the presence of oligodendroglioma-like cellular components, an infiltrative growth pattern, and intense CD34 immunopositivity

**Fig. 2:**
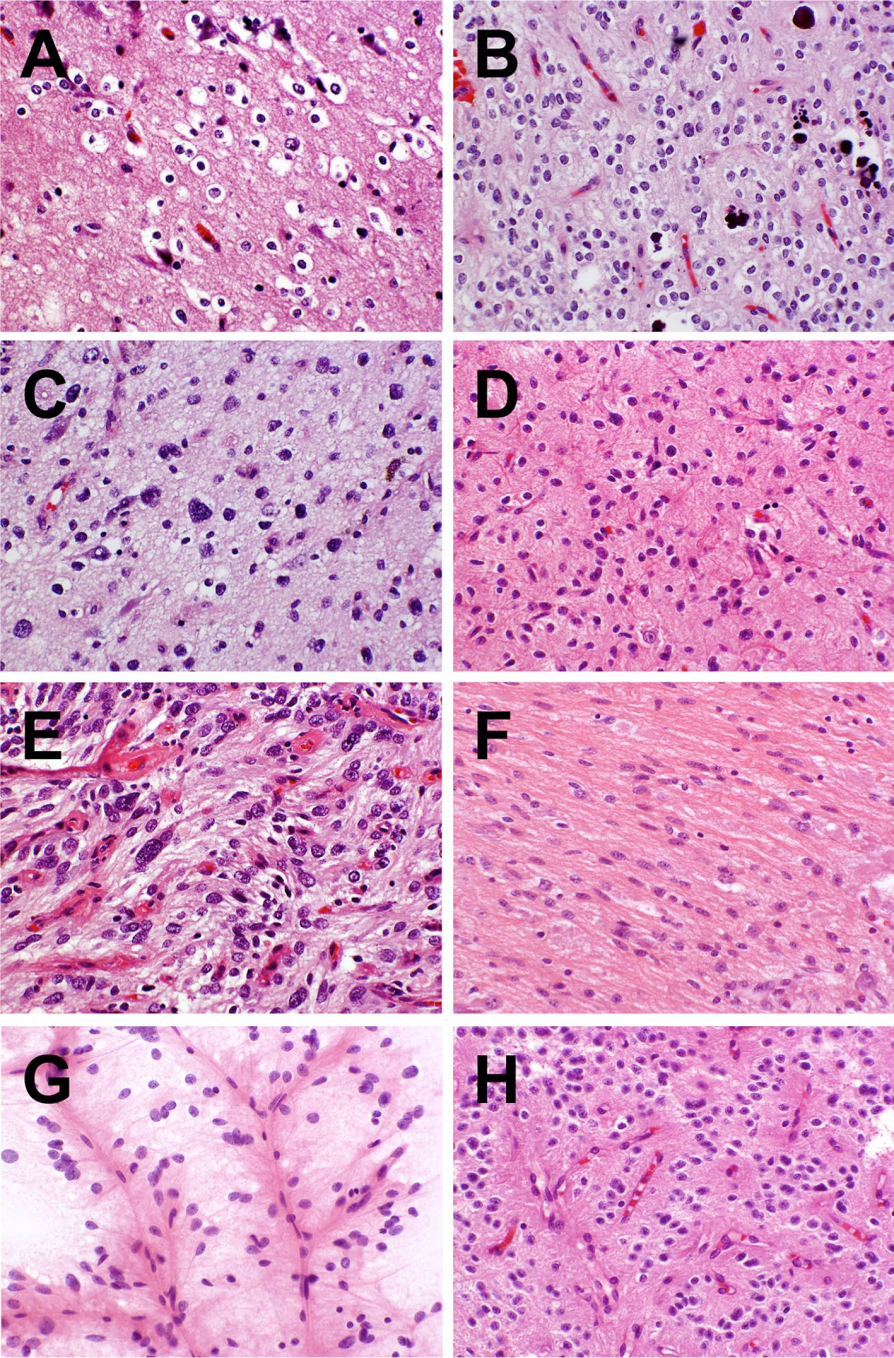
Morphological features of PLNTY. Oligodendroglial (**a**, **b**), fibrillary astrocytic (**c**, **d**), and spindled astrocytic (**e**, **f**) features as seen in cases 3 (**a**, **c**, **e**) and 6 (**b**, **d**, **f**). Vague perivascular pseudorosetting (**g**, **h**) as seen in case 5 by smear preparation (**g**) as well as sectioning (**h**)

All ten tumors in our sample set evidenced infiltrative growth patterns (many also having more compact regions) and harbored oligodendroglioma-like components (Fig. 2a, b), these constituting the nearly exclusive population in one example (case 1) and dominating three other lesions (cases 5, 6, and 7). Exhibiting both intra- and inter-tumoral heterogeneity, these ranged from classically oligodendrocyte-like elements having uniformly small and perfectly rounded nuclei with conspicuous perinuclear halo effects to components displaying considerable variation in nuclear size, more oval or spindled nuclear contours, nuclear membrane wrinkling, grooving, and occasional intranuclear pseudoinclusions. In some fields, the equidistant distribution of tumor cells in relation to stromal capillaries resulted in a vague or more decided pseudorosetting (Fig. 2g, h), intraoperative smear preparations potentially revealing pseudorosetted attachment of neoplastic cells to blood vessels as well. Except case 1 all tumors harbored elements of patently astrocytic or cytologically ambiguous aspect also (Fig. 2c, f). The former included fibrillary, spindled, and pleomorphic populations of varying cell density. Calcifications were identified in nine of ten cases, ranging from discrete calcospherules to confluent calcific masses with osseous metaplasia. Perivascular lymphocytic infiltration was conspicuous only in case 1 and possibly related to prior electrode grid placement. Notably absent were gemistocytic elements, Rosenthal fibers, eosinophilic granular bodies, myxoid microcysts, dysmorphic neuronal/ganglion cell forms, neurocytic or ependymal rosettes, microvascular proliferation, and necrosis. In no case was a “specific glioneuronal element” of dysembryoplastic neuroepithelial tumor type identified. Rare mitoses were found in cases 6 and 7, the mitotic rate in other lesions being essentially nil. Two lesions (cases 1 and 4) were associated with anomalous neuronal clustering in the regional cerebral cortex.

**Fig. 3:**
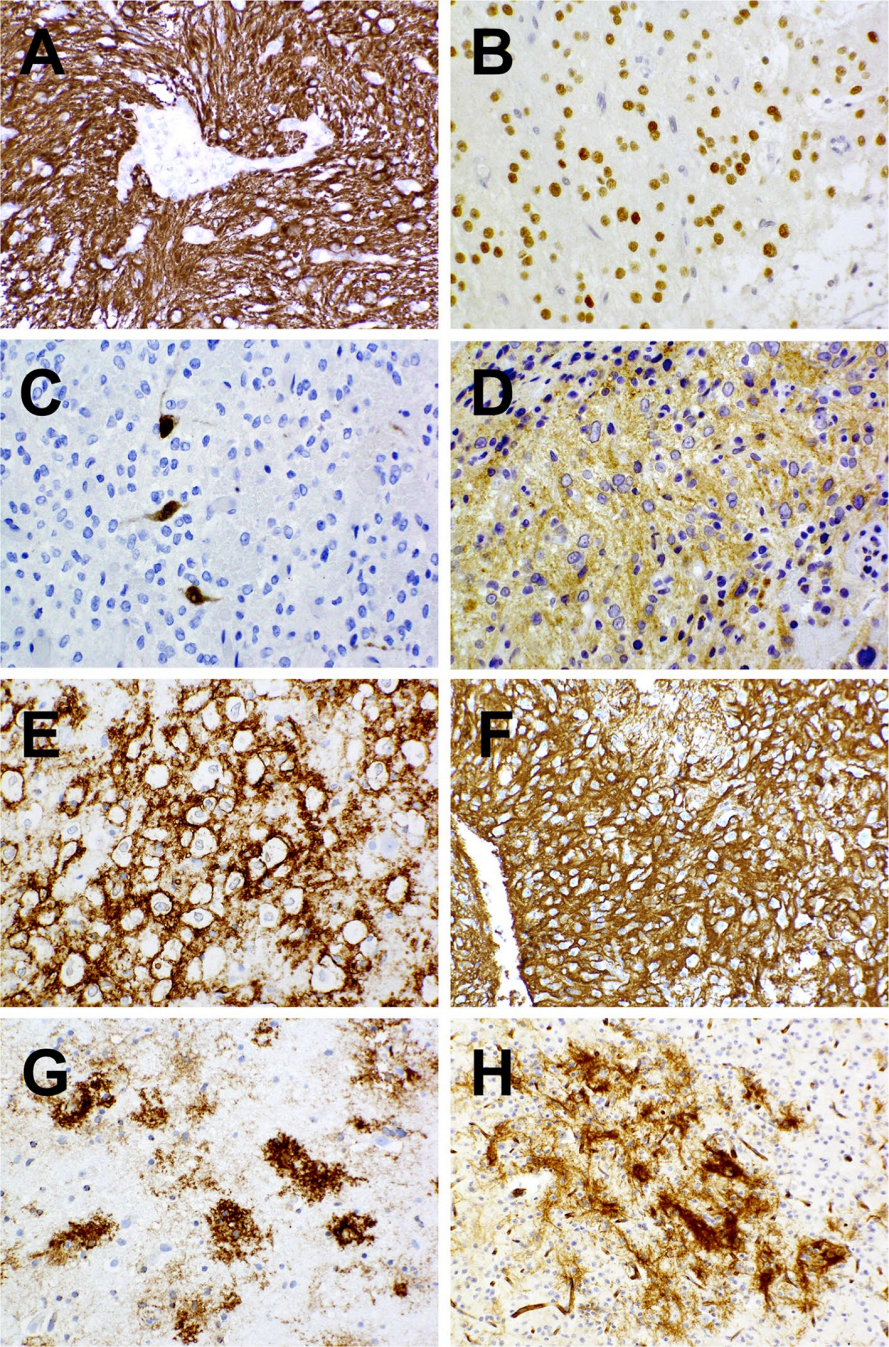
Immunohistochemical features of PLNTY. Strong labeling for GFAP as seen in case 3 (**a**), OLIG2 positivity as seen in case 2 (**b**), negativity for HuC/HuD as seen in case 6, and expression of BRAF V600E as seen in case 3. Diffuse strong expression of CD34 in both tumor cells (**e**, **f**) and peripherally associated ramified neural elements (**g**, **h**) as seen in case 3 (**e**, **g**) and case 6 (**f**, **h**)

All ten cases assessed exhibited expression of glial fibrillary acidic protein (GFAP; Fig. 3a), though this was often focal or patchy and only weak cytoplasmic labeling characterized three lesions. Areas of pseudorosette formation demonstrated GFAP-immunoreactive cell processes converging on vessel walls. All ten cases studied also manifested both intense and often widespread tumor cell expression of CD34 as well as the presence in regional cortex of ramified, CD34-expressing neural elements in large numbers (Fig. 3e, h). In some cases, regions of striking CD34 expression by neoplastic cells abruptly gave way to areas in which tumor cells of identical histological appearance were entirely CD34-nonreactive. All nine cases studied exhibited widespread OLIG2 expression (Fig. 3b). Three of ten cases (1, 2, and 3) demonstrated BRAF V600E expression that was relatively weak (Fig. 3d), though mutation was confirmed on molecular assessment (see below). One case (10) manifested weak matrix labeling for synaptophysin that could not be distinguished from background neuroparenchymal reactivity. All ten cases were entirely negative for expression of HuC/HuD (Fig. 3c), NeuN, chromogranin, and IDH1 (R132H), none showing dot- or ring-type EMA labeling. All seven cases assessed demonstrated retained expression of ATRX. MIB-1/Ki-67 labeling activity was negligible in eight of ten cases, being well below 1%. Cases 6 and 7 harbored microscopic foci in which he MIB-1/Ki-67 labeling indices rose to 5 and 3%, respectively.

None of the ten tumors assessed exhibited evidence of chromosome 1p or 19q deletion by fluorescence in situ hybridization (FISH). Case 1 demonstrated 3–4 copies of 1p36/1q36 and 19p13/19q13 in 20% and 56% of cells, respectively. Case 2 harbored 3-4 copies of 1p36/1q36 and 19p13/19q13 in 17 and 40% of cells, respectively. Three copies of 19p13/19q13 were found in 74% of cells in case 10.

### PLNTYs are characterized by molecular abnormalities involving MAP kinase pathway constituents

To identify potential oncogenic driver events in PLNTYs, we subjected our sample set to both directed and unsupervised molecular profiling. Given the frequency with which molecular abnormalities involving BRAF and other MAP kinase pathway components feature in low-grade epileptogenic brain tumors of childhood [21]—particularly pilocytic astrocytoma, pleomorphic xanthoastrocytoma (PXA), and ganglioglioma—we reasoned that PLNTYs might harbor similar events. First, we employed validated Sequenom-based genotyping for BRAF V600E, the most common cancer-associated BRAF mutation. Eight of the ten cases in our sample set had sufficient material for DNA-based analyses (Table 1), and within this subset we identified three tumors with BRAF V600E mutations (Table 1). We subsequently confirmed our genotyping findings using mutation-specific immunohistochemical staining (Fig. 3d).

**Fig. 4:**
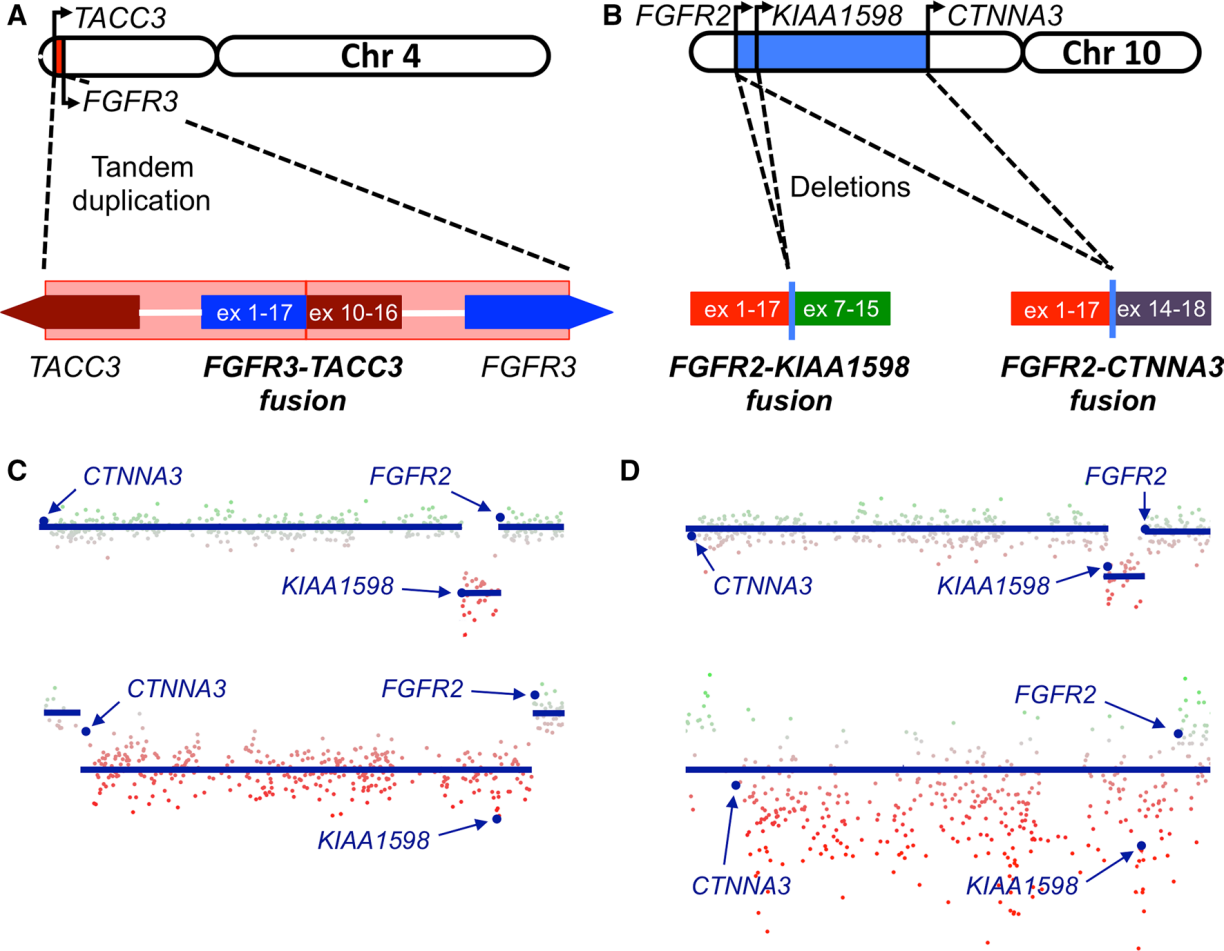
FGFR fusions in PLNTY. Schematics showing the tandem duplication and deletion events resulting in FGFR3-TACC (**a**), FGFR2-KIAA1598 (**b**), and FGFR2-CTNNA3 (**b**) fusions. **c** Copy number profiles from chromosome 10 showing causative deletion events for FGFR2-KIAA1598 (*upper*) and FGFR2-CTNNA3 (*lower*) fusions in two ArcherDx-confirmed cases (5 and 7).* Red* and* green* indicate relative copy number loss and gain respectively. The genomic positions of *CTNNA3*, *KIAA1598*, and *FGFR2* are indicated. **d** Both fusion events are recapitulated in two additional cases identified in the DFKZ database. In the case of the likely FGFR2-CTNNA3 fusion (*lower*), noise precludes definitive segmentation of the deletion event (*blue line*)

We also employed RNA-seq-based methodology (ArcherDx) to identify fusion transcripts in six of our samples, with the initial aim of assessing whether the KIAA1549-BRAF fusion event commonly found in pilocytic astrocytomas [4, 22, 30, 38] exhibits similar prevalence in PLNTYs. While we were unable to demonstrate KIAA1549-BRAF transcripts in any profiled tumors, we identified fusions involving fibroblast growth factor receptors, FGFR2 and FGFR3, in three cases (Fig. 4a, b; Table 1). Notably, FGFR fusions were mutually exclusive with BRAF V600E mutations across our sample set. Two of the identified FGFR fusions, FGFR3-TACC3 and FGFR2-KIAA1598, were initially reported in earlier profiling studies of cholangiocarcinoma, GBM, and pediatric low-grade glioma [16, 18, 39, 48]. Moreover, FGFR3-TACC3 has been validated as an oncogenic driver in multiple glioma-relevant disease model systems [39].

FGFR fusions are thought to drive enhanced downstream signaling through MAP kinase pathway effectors by way of homodimerization and autophosphorylation, a process mediated by coiled coil domains resident in their respective fusion partners (e.g., TACC3, KIAA1598) [16, 39]. The third fusion transcript identified in our sample set, which is novel, pairs FGFR2 with exons 14–18 of CTNNA3. Interestingly, while CTNNA3 does not harbor coiled coils per se, exons 14–18 encode the entirety of its C-terminal dimerization domain required for the stabilization of cell–cell junctions [34]. In this way, FGFR2-CTNNA3 likely functions in the same manner as other previously reported FGFR fusions. Finally, copy number profiling derived from Infinium methylation analysis (see below) demonstrated that FGFR3 and FGFR2 fusions derived from tandem duplication and deletion events, respectively, consistent with prior reports (Fig. 4a, d). Moreover, these data revealed an additional likely FGFR2-KIAA1598 fusion event in a tumor that had not undergone ArcherDX analysis (Table 1, case 6). No additional recurrent copy number aberrations were identified (FIG. S1).

### Genome-wide methylation profiling segregates PLNTYs from other pediatric low-grade neuroepithelial tumors

**Fig. 5:**
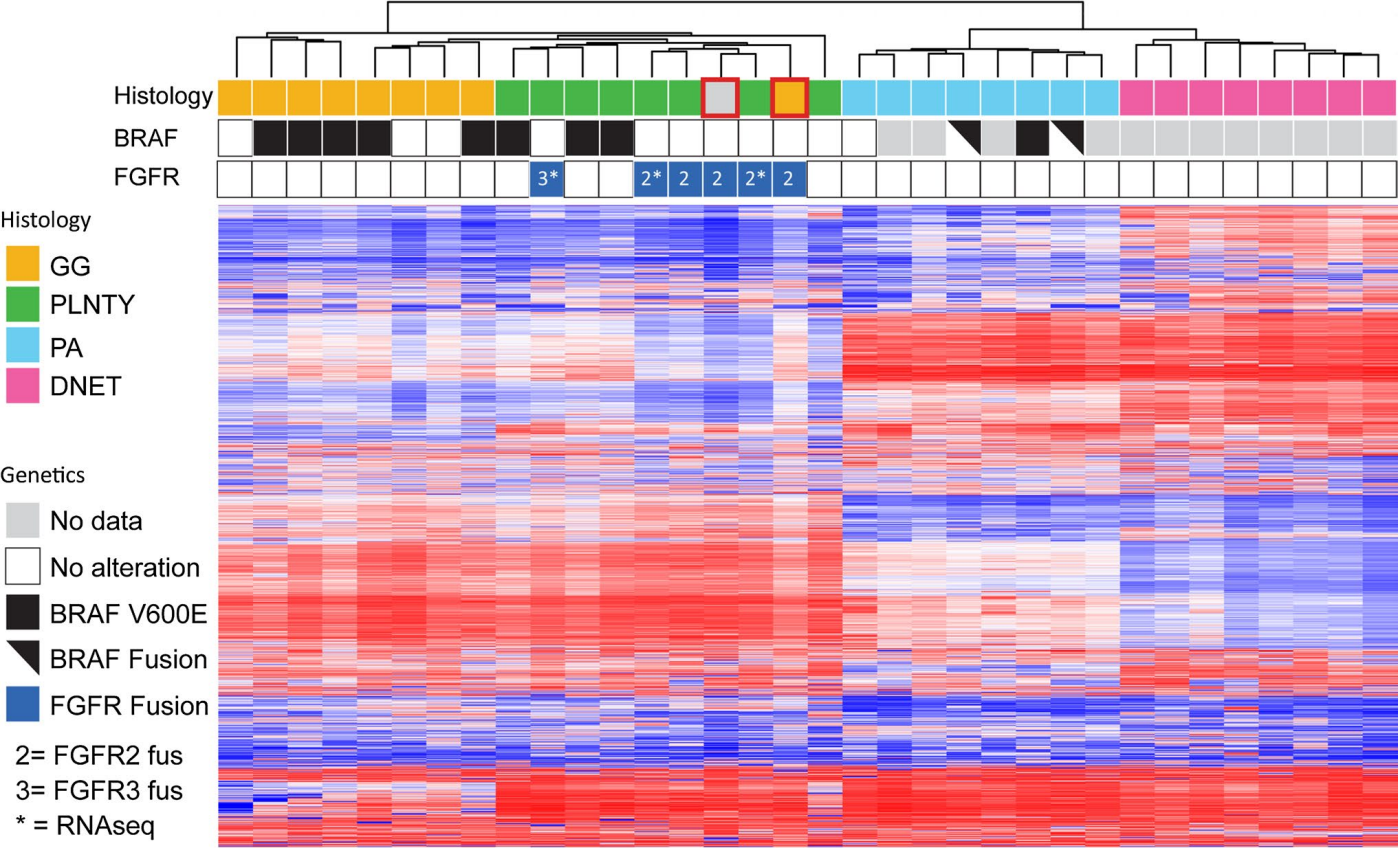
Global methylation profiling delineates PLNTY from other epileptogenic tumors of children and young adults. Heat map (*red* increase methylation, *blue* decreased methylation) showing distinct CpG methylation signature for PLNTY. Histopathologic and genetic correlates are shown. *GG* ganglioglioma, *PA* pilocytic astrocytoma, *DNET* dysembryoplastic neuroepithelial tumor, *LGG NOS* low-grade glioma, not otherwise specified. *Red squares* indicate DFKZ samples not in the original MSKCC cohort

To better determine the precise molecular classification of PLNTYs and gain insight into their histogenesis, we performed genome-wide DNA methylation analysis on eight samples in our cohort using the Infinium MethylationEPIC platform. Numerous studies have demonstrated the utility of methylation profiling in establishing robust molecular taxonomies that often transcend conventional histopathologic designations [20, 27, 31, 40, 41], a process greatly facilitated by a large existing repository of brain tumor array data maintained at the German Cancer Research Center (DFKZ). Examining our methylation findings in the context of this database, we found that PLNTYs exhibited a distinct signature, most closely related to gangliogliomas, with similarities to pilocytic astrocytomas and DNETs as well (Fig. 5). Importantly, *BRAF* alterations (V600E mutations and KIAA1549-BRAF fusions) were distributed across multiple diagnostic groups—PLNTY, ganglioglioma, and pilocytic astrocytoma—each of which exhibited a distinct methylation signature. More distant associations with other neuroepithelial tumor entities, including PXA, IDH-mutant diffuse astrocytoma and oligodendroglioma, and pediatric low-grade glioma with MYB/MYBL1 alterations were revealed by t-sne scatterplotting (FIG. S2). Our analysis also revealed two additional cases among previously profiled tumors, histopathologically classified as ganglioglioma and “low-grade glioma”, which clustered with the PLNTY methylation signature. Interestingly, these tumors harbored likely FGFR2 fusions by copy number analysis (Fig. 4c, d). Taken together, these findings suggest that PLNTYs are a distinct biological entity related to other low-grade neuroepithelial neoplasms primarily affecting children and young adults.

## Discussion

LGNTs of children and young adults are a heterogeneous group of neoplastic entities whose fundamental identities reflect both molecular and histogenic factors. In recent years, comprehensive genomic profiling in large patient cohorts has confirmed the biological distinctiveness of some well-established histopathologic variants, such as pilocytic astrocytoma, PXA, and angiocentric glioma [3, 4, 22, 30, 31, 33, 38, 48]. However, other morphological subtypes, including oligodendroglial tumors, have exhibited more variable molecular features, implying the underlying existence of multiple distinct disease entities. In this study, we sought to characterize a specific oligodendroglioma-like LGNT with characteristic clinical behavior, localization, and histopathology. We found that these tumors form a cohesive biological subgroup characterized by intense CD34 expression, molecular abnormalities involving MAPK signaling, and a distinct DNA methylation signature.

The designation of PLNTYs as “polymorphous” is meant to acknowledge the fact that, while all harbored components prompting consideration of oligodendroglioma in the differential diagnosis, most also contained populations of patently astrocytic or ambiguous appearance and several manifested foci of vague or more fully developed perivascular pseudorosetting that raised question of ependymocytic differentiation. Consistent expression of GFAP and OLIG2 in the absence of dot- or ring-type EMA labeling and failure to express neuronal “markers” would argue for these lesions fundamentally representing non-ependymal gliomas. However, we think it prudent to reserve final judgment on their fundamental differentiating capacity and so refer to these generically as “neuroepithelial”. The issue of their molecular relationship to gangliogliomas is further addressed below. There can be little doubt that the neoplasms of the PLNTY group are part of a larger family of tumors reported as diffuse astrocytomas or oligodendrogliomas of pediatric type having MAPK pathway-activating genetic abnormalities [31, 48]. Similarly, the lesions we describe as PLNTYs would appear to lie among CD34-expressing neoplasms constituting a subset of growths for which Blumcke and colleagues have proposed the designation “long-term epilepsy associated tumors” (LEATs) [7]. Whatever terminology is eventually employed, the designation of PLNTYs should clearly segregate them from the ineluctably recurring and genetically distinct gliomas of infiltrating type encountered in generally older patients.

A constant and striking feature of our tumors was intense, frequently widespread expression of CD34 by tumor cells and by ramified neural elements in the regional cerebral cortex. In fact, it was this immunohistochemical observation, along with the consistent failure of the tumors in this series to manifest labeling for IDH1 R132H or chromosome 1p/19q codeletion, despite their oligodendrogliomatous appearances, that prompted us to consider these as distinct from the infiltrating gliomas of later life and to initiate additional molecular studies. While the immunohistochemical labeling of some glioblastomas for CD34 is recognized [19, 25], this phenomenon is generally foreign to infiltrating astrocytomas of lower grade and to neoplasms of the oligodendroglioma group [2, 5, 6, 10, 13–15, 28, 29, 32, 36, 43, 45–47]. Only one of the many studies that have examined CD34 expression in gliomas found tumoral (as opposed to endothelial) immunoreactivity in lesions classified as WHO grade II or III astrocytomas, oligodendrogliomas or oligoastrocytomas [8]. Of note, and emphasized by the authors of this single outlier, such reactivity was confined to examples deriving from the temporal lobes of patients with chronic and refractory seizures whereas “tumor samples derived from non-epileptic patients with lesions outside the temporal lobe never showed CD34 -immunoreactive neural cells” [8]. It is likely that at least some of these ostensibly conventional gliomas represented neoplasms of the type we describe.

In addition to distancing lesions of the PLNTY group from conventional “adult-type” gliomas of the infiltrating variety (a matter of great practical import given that the latter are rarely curable by surgical or adjuvant means, are prone to progression and eventually fatal in the great majority of cases), shared CD34 expression may well reflect a link between PLNTYs and other tumors long recognized for their association with chronic epilepsy in young subjects. Specifically, expression of CD34 is a common attribute of pediatric gangliogliomas [8–10, 17] and PXAs [17, 35], having also been recorded by some observers in the setting of DNET [42, 44]. There is, furthermore, a strong association of these epileptogenic tumors with the presence in regional cortical tissues of the ramified, CD34- expressing neural elements observed in complex with each of our cases. Foreign to the normal human brain, these have also been found in association with glioneuronal microhamartomas (“hamartia”) and some forms of cortical dysplasia [8, 17]. The transient expression of CD34 in the murine neural tube [12, 24] has elicited speculation that these ramified cells represent developmentally arrested/dysregulated neural progenitors that testify to regional dysontogenesis and that may give rise to the CD34-expressing tumors with which they are identified [8–10]. The superficial positioning of the neoplasms reported here, their predilection for the temporal lobes, presentation in the young, association with chronic epilepsy, general indolence, CD34 expression, MAPK pathway-activating genetic lesions and methylation profiles would all support a link to the pediatric ganglioglioma and other “developmental” tumors. In fact, it could be argued that the tumors of our PLNTY group represent “lopsided” variants of ganglioglioma in which neuronal components and even immunophenotypic evidence of neuronal differentiation may not be demonstrable, though whether these ostensibly infiltrative lesions are as biologically stable and surgically curable as classical gangliogliomas remain to be seen.

Using focused molecular profiling, we found that nearly all of the PLNTYs harbored either BRAF V600E mutations or fusion events involving FGFR2/FGFR3. These abnormalities occurred in a mutually exclusive fashion, consistent with the notion that shared downstream molecular sequelae fundamentally drive the pathogenesis of PLNTYs. Indeed, both V600E-mutant BRAF monomers and FGFR fusion chimeras have been shown to activate MAPK signaling, the latter by way of constitutive receptor dimerization [16, 39], a mechanism likely operative in all such cases in our sample set (see above). That being said, abnormal MAPK pathway activation, by either BRAF V600E or FGFR fusion, is hardly specific to PLNTYs as a transforming molecular event. BRAF V600E has been widely implicated in the pathogenesis of LGNTs, with particularly high rates of incidence in gangliogliomas and PXAs [37]. Similarly, recent work has identified frequent FGFR fusion events in DNETs, and diffusely infiltrating pediatric gliomas exhibiting either astrocytic or oligodendroglial histopathology [31]. As stated above, we believe that PLNTYs likely represent a subset of this latter group. BRAF V600E mutations and FGFR fusions are even found in a small minority of glioblastomas [11]. Notably, none of the tumors in our sample cohort harbored KIAA1549-BRAF fusions, which are commonly associated with pilocytic astrocytomas and were recently reported in a small set of two neoplasms classified as “pediatric oligodendrogliomas” [23]. The broad spectrum of brain tumors exhibiting MAPK pathway activation attests to its importance as an oncogenic mechanism, while also indicating that its precise transformative consequences are highly dependent on biological context.

Over the past several years, genome-wide DNA methylation profiling has become an influential tool informing the classification of primary brain tumors. For instance, methylation signatures have been repeatedly utilized to establish endogenous subclasses within morphologically based disease entities, most notably for glioblastoma and medulloblastoma [20, 41]. Methylation profiling has also facilitated more appropriate tumor classification within poorly defined histopathologic groupings. In a recent study of supratentorial primitive neuroectodermal tumors (PNETs), methylation arrays delineated the vast majority of sampled neoplasms into other more well-established diagnostic categories, including glioblastoma and ependymoma [40]. In these respects, specific patterns of CpG island methylation capture the biological distinctiveness of a given neoplastic process. In our analysis, PLNTYs exhibited a unique DNA methylation signature whose distinctiveness even allowed the identification of additional cases from a much larger sample cohort that had already been subjected to profiling. Interestingly, the PLNTY methylation signature was most closely related to those of other, morphologically diverse LGNTs—gangliogliomas, pilocytic astrocytomas, and DNETs—characterized by MAPK pathway activation. This finding implies that the unique aspects of the PLNTY methylation signature relate to issues of histogenesis and differentiation state rather than the primary oncogenic driver mechanism.

Applying our findings to the diagnostic setting, several considerations should be kept in mind. While the demonstration of aberrant CD34 expression by neoplastic and ramified neural elements certainly suggests PLNTY in the appropriate context, calling into serious question the diagnosis of any conventional glioma of infiltrating type, it would be premature to conclude that this finding obviates the need for assessments of *IDH1/2* mutation and chromosome 1p/19q codeletion status in the evaluation of neurosurgical specimens. In a similar vein, CD34 expression cannot be proposed as a surrogate marker of MAPK pathway activation at this time. Candidate cases should be studied for *BRAF* and *FGFR2/3* abnormalities and an integrated diagnosis rendered on the basis of the histologic, immunophenotypic, and molecular genetic features. In fact, we believe that ostensibly infiltrating glial neoplasms deriving from young individuals (especially those posing as chromosome 1p/19q non-codeleted oligodendrogliomas) should be assessed in similar fashion irrespective of CD34 expression status, as the CD34-labeling tumors we describe may constitute but a subset of fundamentally unified neuroepithelial neoplasms sharing a spectrum of MAPK pathway-activating genetic lesions. In this connection, we would point out that the “pediatric oligodendrogliomas” (and other low-grade gliomas of the young) reported to date as displaying BRAF and FGFR abnormalities seem not to have been systematically studied for CD34 expression [23, 31, 48].

In summary, we report the characterization of PLNTY, a molecularly distinct LGNT that likely represents a significant subset of oligodendroglioma-like tumors arising in pediatric populations. PLNTYs exhibit an almost invariably benign clinical course and appear to be well controlled by gross total resection. Moreover, their driving molecular alterations, which uniformly activate MAPK signaling, are potentially targetable with existing small molecular inhibitors, providing additional therapeutic options for affected patients.

## Electronic supplementary material

Below is the link to the electronic supplementary material.

Copy number traces derived from global methylation profiling arrays for MSKCC samples corresponding to cases 1-8 along with two PLNTY cases identified from the DFKZ patient cohort (DFKZ 1-2) (TIFF 24890 kb)

t-SNE plot showing DNA methylation differences between tumor groups, based on the 5,000 most differentially methylated CpG probes across the cohort (standard deviation). PLNTY tumors form distinct groups that are highly related to ganglioglioma, while clearly different from a variety of other pediatric and adult brain tumors. A_IDH, IDH-mutant 1p19q intact (astrocytic) glioma; O_IDH, IDH-mutant 1p19q-codeleted (oligodendroglial) glioma; GG, ganglioglioma; PA, (cortical) pilocytic astrocytoma; DNET, dysembryoplastic neuroepithelial tumor; PXA, pleomorphic xanthoastrocytoma; LGG_MYB, low-grade diffuse glioma with alterations of MYB/MYBL1 (TIFF 20714 kb)
