# Supplementary figures and images for: Polymorphous low-grade neuroepithelial tumor of the young (PLNTY): an epileptogenic neoplasm with oligodendroglioma-like components, aberrant CD34 expression, and genetic alterations involving the MAP kinase pathway
Source: Acta Neuropathol. 2016 Nov 3;133(3):417–29. doi: 10.1007/s00401-016-1639-9 (PMC5325850; doi:10.1007/s00401-016-1639-9)

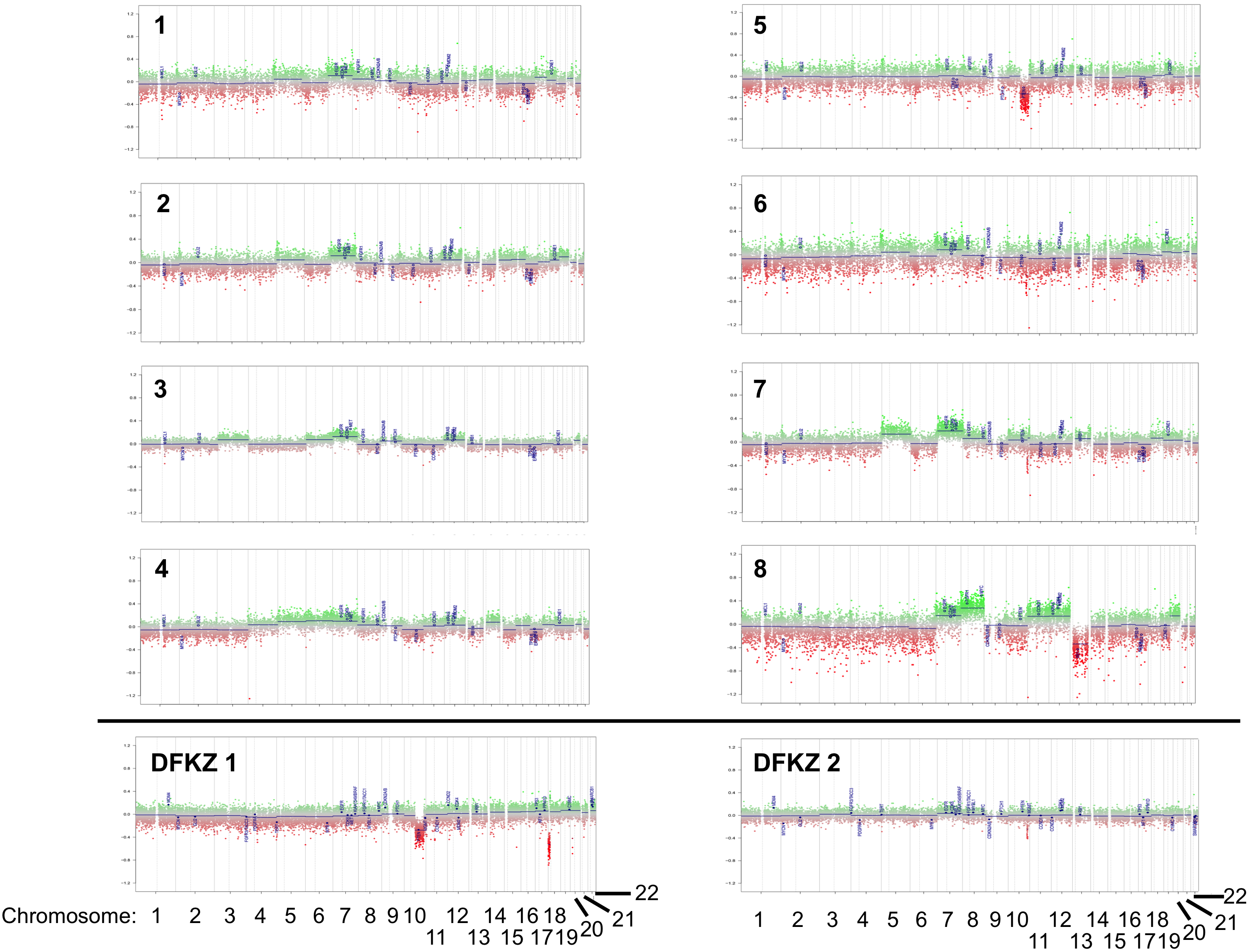

Supplement: Supplementary file 1 — Copy number traces derived from global methylation profiling arrays for MSKCC samples corresponding to cases 1-8 along with two PLNTY cases identified from the DFKZ patient cohort (DFKZ 1-2) (TIFF 24890 kb) [file 401_2016_1639_MOESM1_ESM.tiff]

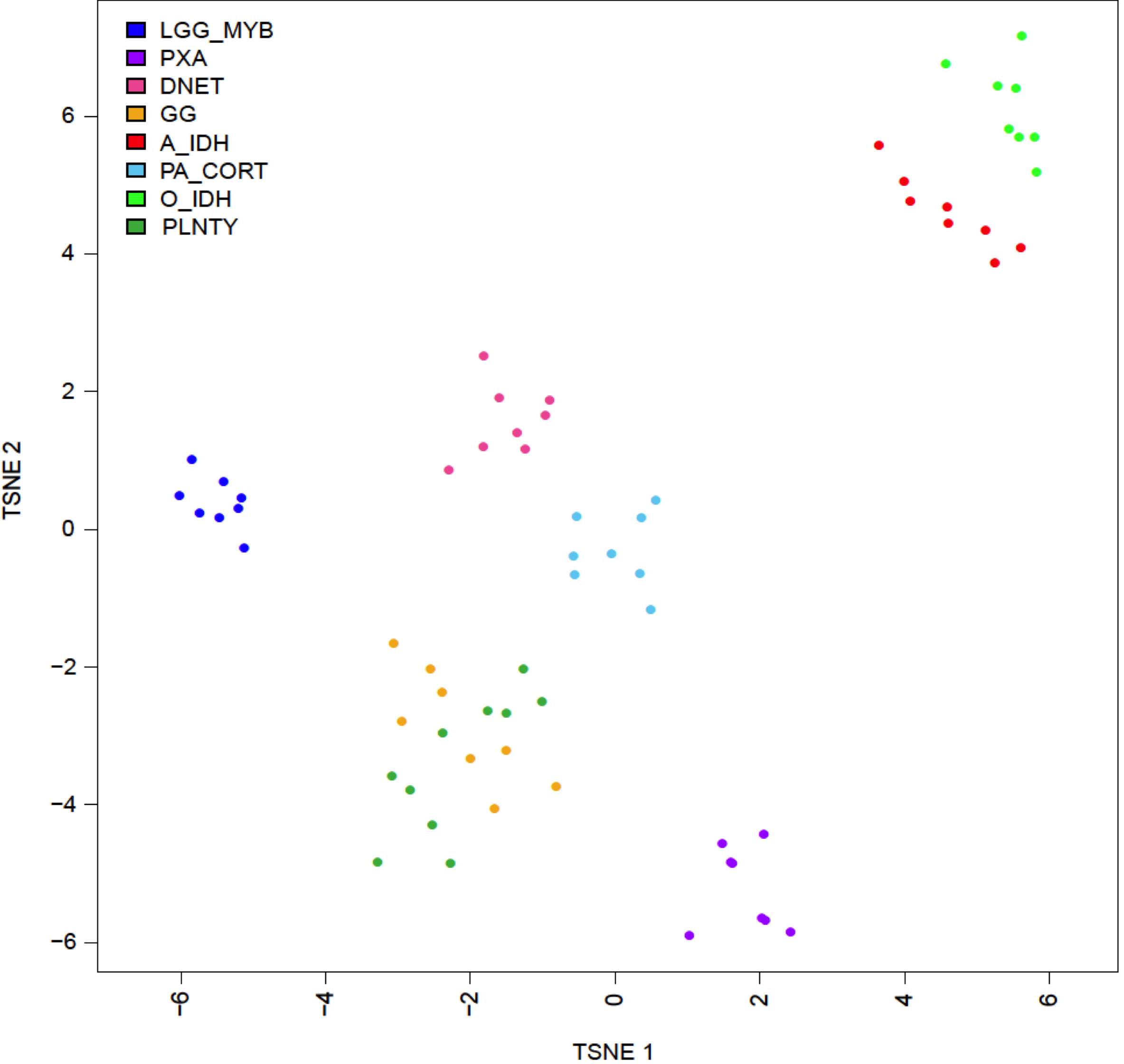

Supplement: Supplementary file 2 — t-SNE plot showing DNA methylation differences between tumor groups, based on the 5,000 most differentially methylated CpG probes across the cohort (standard deviation). PLNTY tumors form distinct groups that are highly related to ganglioglioma, while clearly different from a variety of other pediatric and adult brain tumors. A_IDH, IDH-mutant 1p19q intact (astrocytic) glioma; O_IDH, IDH-mutant 1p19q-codeleted (oligodendroglial) glioma; GG, ganglioglioma; PA, (cortical) pilocytic astrocytoma; DNET, dysembryoplastic neuroepithelial tumor; PXA, pleomorphic xanthoastrocytoma; LGG_MYB, low-grade diffuse glioma with alterations of MYB/MYBL1 (TIFF 20714 kb) [file 401_2016_1639_MOESM2_ESM.tiff]
